# Transcription factor expression levels and environmental signals constrain transcription factor innovation

**DOI:** 10.1099/mic.0.001378

**Published:** 2023-08-16

**Authors:** Matthew J. Shepherd, Mitchell Reynolds, Aidan P. Pierce, Alan M. Rice, Tiffany B. Taylor

**Affiliations:** ^1^​ Milner Centre for Evolution, Department of Life Sciences, University of Bath, Claverton Down, Bath BA2 7AY, UK; ^2^​ Division of Evolution and Genomic Sciences, School of Biological Sciences, University of Manchester, Manchester, UK

**Keywords:** experimental evolution, gene regulatory networks, *Pseudomonas fluorescens*, transcription factor, rhamnose inducible expression

## Abstract

Evolutionary innovation of transcription factors frequently drives phenotypic diversification and adaptation to environmental change. Transcription factors can gain or lose connections to target genes, resulting in novel regulatory responses and phenotypes. However the frequency of functional adaptation varies between different regulators, even when they are closely related. To identify factors influencing propensity for innovation, we utilise a *Pseudomonas fluorescens* SBW25 strain rendered incapable of flagellar mediated motility in soft-agar plates via deletion of the flagellar master regulator (*fleQ*). This bacterium can evolve to rescue flagellar motility via gene regulatory network rewiring of an alternative transcription factor to rescue activity of FleQ. Previously, we have identified two members (out of 22) of the RpoN-dependent enhancer binding protein (RpoN-EBP) family of transcription factors (NtrC and PFLU1132) that are capable of innovating in this way. These two transcription factors rescue motility repeatably and reliably in a strict hierarchy – with NtrC the only route in a ∆*fleQ* background, and PFLU1132 the only route in a ∆*fleQ*∆*ntrC* background. However, why other members in the same transcription factor family have not been observed to rescue flagellar activity is unclear. Previous work shows that protein homology cannot explain this pattern within the protein family (RpoN-EBPs), and mutations in strains that rescued motility suggested high levels of transcription factor expression and activation drive innovation. We predict that mutations that increase expression of the transcription factor are vital to unlock evolutionary potential for innovation. Here, we construct titratable expression mutant lines for 11 of the RpoN-EBPs in *P. fluorescens *. We show that in five additional RpoN-EBPs (FleR, HbcR, GcsR, DctD, AauR and PFLU2209), high expression levels result in different mutations conferring motility rescue, suggesting alternative rewiring pathways. Our results indicate that expression levels (and not protein homology) of RpoN-EBPs are a key constraining factor in determining evolutionary potential for innovation. This suggests that transcription factors that can achieve high expression through few mutational changes, or transcription factors that are active in the selective environment, are more likely to innovate and contribute to adaptive gene regulatory network evolution.

## Introduction

Propensity for innovation is a key determinant of evolvability – the ability to rapidly generate heritable phenotypic variation – in living organisms [1]. Alterations to transcription factor binding specificities and activity levels can facilitate evolution of regulatory connections [2, 3], which generate phenotypic variation and novelty that can provide selective advantages under changeable environmental conditions [4–8]. However, propensity for innovation varies between transcription factors [9], and whilst features influencing evolvability of transcription factor bindings sites have been investigated [10, 11], causes of variation in transcription factor evolvability remain poorly defined. To understand how novelty in regulatory systems evolves, we must identify intrinsic and environmental factors that determine rates of evolutionary innovation in these regulatory proteins.

A key property for evolvability in a transcription factor is its ability to gain novel connections, which often involves gain of promiscuous activity. For a transcription factor, this constitutes gain of illicit or non-canonical regulatory interactions, and is a key factor in revealing a transcription factor to selection and for evolutionary innovation to occur [12, 13]. These interactions typically bear no physiological significance, but under the right selective conditions can become advantageous and drive innovation [14, 15]. Previously, we investigated the evolutionary emergence of promiscuity in the RpoN-dependant enhancer binding proteins (RpoN-EBPs) NtrC and PFLU1132 in the soil bacterium *Pseudomonas fluorescens* [2, 16]. We made use of an engineered maladapted gene regulatory network (GRN), where the RpoN-EBP flagellar master regulator FleQ is deleted resulting in loss of flagellar expression and motility. When challenged to rescue motility, NtrC and PFLU1132 evolved promiscuous activity to drive flagellar gene expression. Whilst this was in part due to the shared 3D structural homology, a result of shared ancestry [17] between FleQ, NtrC and PFLU1132 that permits DNA binding without the need for mutation to the DNA binding domain, we also found that high gene expression and high levels of activation in these transcription factors was important for their promiscuous activity and innovation [2]. These properties likely aid low-affinity promiscuous interactions to occur by providing an excess of a highly activated transcription factor that can saturate its native regulatory interactions and begin to engage in additional promiscuous regulatory activities.

The expression and activation levels of a transcription factor are determined by the architecture and connectivity of the GRN it sits within [18]. Our previous findings therefore raise the possibility that pre-existing GRN architecture may significantly bias and constrain transcription factor evolution. For example, some transcription factors may face significant obstacles to gaining high expression levels. Many are negative autoregulators (41 % of transcription factors in *E. coli *) [19], where multiple precise promoter mutations [20] would be needed to increase expression level. Conversely, some transcription factors may be ideally suited to gaining high expression, through virtue of positive autoregulation [21–23], where loss of a negative repressor can easily lead to runaway feedback driving high transcription factor expression [24–26].

Similarly, there will be variation in how easily a transcription factor may gain a hyperactivated state. This again depends on the signalling connectivity of the transcription factor within the GRN. Bacterial regulatory networks will sense and transduce internal or environmental stimuli [27], commonly through two-component systems (TCS’s) where a sensor-kinase detects a signal and phosphorylates a phosphoacceptor receiver (REC) domain on a cognate transcription factor [28]. Alternatively, transcription factors can possess receiver domains that: directly bind small molecule signals, are bound by a protein inhibitor, or control sub-cellular localisation to determine activation [29]. Mutations to TCS kinases are frequently observed to drive adaptation in regulatory systems [30–32], and transcription factors that respond to particularly active or mutable cellular systems may more easily become hyperactivated.

This study aims to investigate the combined role of these intrinsic (transcription factor expression, activity, and connectivity with the GRN) and extrinsic (presence of signals in the environment or from other cells) factors in determining evolutionary pathways and trajectories followed during phenotypic adaptation, and in particular how variation in transcription factor gene expression can impact adaptive outcomes. To do this we make use of a previously characterised model system of transcription factor evolution. In our model, we have identified two RpoN-EBPs – NtrC and PFLU1132 – capable of rescuing flagellar motility via promiscuous activity. There are 19 other RpoN-EBPs that could also theoretically achieve this (33) due to sharing structural homology with FleQ, however none are observed to do so in previous LB or M9 soft agar evolution experiments [2, 16, 34, 35]. As we observed that a major constraining factor for PFLU1132 promiscuity was its gene expression level [2], we can hypothesise that gene expression level may be constraining evolutionary innovation through gain of promiscuity in the other RpoN-EBPs. To test this, we engineered titratable expression constructs for 11 of the RpoN-EBP genes encoded by *P. fluorescens* SBW25. This was achieved by introduction of the RpoN-EBP coding sequence downstream of a *rhaSR*-P*rhaBAD* l-rhamnose titratable promoter system [36], and inserting this construct as a single copy into the *P. fluorescens* chromosome using the miniTn7 transposonal insertion system [37]. This allows us to increase expression for each RpoN-EBP into the GRN by addition of l-rhamnose to the growth media, simulating an alternative GRN architecture and environment where the transcription factor is highly expressed. Utilising these expression strains, we set out to identify if any other RpoN-EBPs could rescue motility, and if increased expression of an RpoN-EBP would bias evolutionary outcomes in a flagellar motility rescue experiment.

## Methods

### Strains and culture conditions

All ancestral strains in this study are derived from either *Pseudomonas fluorescens* AR2 (SBW25Δ*fleQ* IS-ΩKm-hah: PFLU2552), or AR2Δ*ntrBC* (constructed previously, [2]). AR2 lacks flagellar master regulator FleQ and possesses a transposon-insertional disruption of the gene *viscB* (PFLU2552), rendering it unable to move via flagellar and flagellar-independent spidery-spreading motility respectively, as detailed previously [16, 38]. All routine culturing of strains were cultured on lysogeny broth (LB; Miller) media at 27 °C. *Escherichia coli* strains for cloning were cultured on LB media at 37 °C. For experiments using M9 minimal media, the follow recipe was used: 0.2 % w/v Glucose, 0.1 mM CaCl_2_, 2 mM MgSO_4_, 1× M9 salts (33.7 mM Na_2_HPO_4_, 22 mM KH_2_PO_4_, 8.55 mM NaCl, 9.35 mM NH_4_Cl). In some cases alternatives were used as the sole carbon or nitrogen sources, in which the Glucose or NH_4_Cl were omitted respectively.

### RpoN-EBP expression system construction

Inducible expression constructs for a panel of RpoN-EBPs were constructed in the AR2 genetic background. The RpoN-EBP ORF was amplified by PCR and a strong ribosome binding site (stRBS) introduced upstream with a 7 bp short spacer between the stRBS and the start codon. PCR also introduced restriction enzyme cut sites up and down-stream of the gene, which were used to insert the RpoN-EBP into the multiple cloning site of the miniTn7 suicide vector pJM220 (obtained from the Addgene plasmid repository, plasmid #110559) by restriction-ligation. This positions the RpoN-EBP gene under control of the P*rhaBAD* promoter and downstream of rhaSR, allowing rhamnose-titratable expression of the RpoN-EBP [36] as used to do so previously [2]. This construct was transformed into *E. coli* DH5α by chemical-competence heat-shock. The miniTn7 transposon containing the *rhaSR* genes and the P*rhaBAD*-stRBS-RpoN-EBP construct was then transferred to the *P. fluorescens* chromosome by transposonal insertion downstream of the *glmS* gene via four-parent puddle-mating conjugation [37]. The relevant *E. coli* DH5α pJM220-derived plasmid donor was combined with recipient *P. fluorescens* AR2 strains, transposition helper *E. coli* SM10 λpir pTNS2 and conjugation helper *E. coli* SP50 pRK2073, and Gentamicin resistant *Pseudomonas* selected for on LB supplemented with Gentamicin sulphate and Kanamycin sulphate. Chromosomal insertion of the correct miniTn7 transposon and RpoN-EBP was confirmed by colony PCR. The *rhaSR*-P*rhaBAD*-stRBS-PFLU4895 construct was also transferred to the chromosome by miniTn7 insertion in an AR2Δ*ntrBC* background. Activity of the *rhaSR-*P*rhaBAD* and response to l-rhamnose present in growth media tested using a *rhaSR*-P*rhaBAD*-stRBS-*lacZ* construct in a β-galactosidase activity assay (Fig. S1) as detailed previously [2].

### Motility rescue evolution experiments

Evolutionary rescue of motility was assayed in 0.25 % agar M9 plates as described previously [16, 34]. Pure single colonies were picked and inoculated using a sterile toothpick and incubated at 27 °C. Plates were checked a minimum of twice daily for motility, recording time to emergence. Motile zones were sampled immediately and always from the leading edge. Motile isolates were streaked on LB agar, and a pure colony picked and stored at −80 °C as glycerol stocks of LB overnight cultures. Motility was checked in fresh media supplemented with and without L-rhamnose after isolate to ensure the phenotype was stable (Fig. S2). All subsequent analysis was conducted on these pure motile isolates. Experiment was run for 6 weeks and any replicates without motility after this cut-off recorded as having not evolved.

### Mutation identification by whole genome resequencing, and PCR sanger sequencing

To identify motility rescuing mutations, genomic DNA was extracted from motile strains and their ancestral strain using the Thermo Scientific GeneJET Genomic DNA Purification Kit. Genomic DNA was quality checked using BR dsDNA Qubit spectrophotometry to determine concentration and nanodrop spectrophotometry to determine purity. Illumina NextSeq 2000 sequencing was provided by Seqcenter (Pittsburgh, PA, USA), with a minimum 30× coverage. Returned paired-end reads were further filtered using fastp v0.23.2 [39] with parameters --disable_adapter_trimming --cut_front --cut_tail --cut_mean_quality 30 --qualified_quality_phred 30 --length_required 50. Alignment of quality trimmed reads to the *P. fluorescens* SBW25 reference genome [40] and mutation identification was conducted using breseq [41]. The content of the reference genome was not altered to account for an extra copy of the introduced EBP genes. Due to this, read depth coverage at the native EBP was roughly double the rest of the genome and mutations within one copy of either the introduced or native EBP were at ~50 % allele frequency. Any other reads of the engineered construct that do not align to the reference genome are discarded. Four substitutions/indels were observed in 92–100 % of samples and were ignored in downstream analysis as these mutations were assumed to be present in the AR2 genetic background with respect to the SBW25 reference genome. These mutations were: three small indels at positions 45 881 (86/93 samples), 985 333 (93/93), 3 447 984 (92/93), and an intergenic substitution at position 1 786 536 (93/93). Five SNPs present in both ancestral strains and evolved motile strains are listed in Table S1 but not included in Fig. 3. Additionally, two samples *gcsR*_C1 and Δ*ntrBC*_1 have a *mutS* mutation and subsequently far more additional mutations relative to other samples. These mutations are listed in Table S1 but not included in Fig. 3. For motile isolates where mutations occurred in the RpoN-EBP gene that had been overexpressed, we determined which RpoN-EBP copy the mutation was present in by PCR amplification of the copy present on the *rhaSR*-P*rhaBAD* inducible expression construct with primers binding either side of the multiple cloning site (pJM220 300 bp Forward and Reverse primers). PCR products were cleaned up using the Monarch PCR cleanup kit (NEB), and Sanger sequenced using the service provided by Source Bioscience. Sequences of the introduced EBP copy generated by Sanger sequencing were aligned to the coding sequence of their respective EBP from the reference genome using MAFFT v7.511 [42]. The presence/absence of EBP variants are noted in the ‘EBP copy’ column in Table S1. All primers used in this study are given in Table S2.

### RpoN-EBP protein domains

To identify where mutations affect the protein domains of RpoN-EBPs, the *P. fluorescens* SBW25 proteome was searched for Pfam 35.0 domains [43] using InterProScan v5.59–91 [44].

To count RpoN-EBPs in *Pseudomonas* species, complete *Pseudomonas* genomes were obtained from NCBI RefSeq [45]. A representative strain was selected for species with numerous strains. After ensuring ≥90 % of *Pseudomonadales* marker genes were present and ≤2 % were duplicated using BUSCO [46], 140 genomes remained. The proteomes were searched using InterProScan for ‘Sigma-54 interaction domain (PF00158)’ Pfam signature to identify RpoN-EBPs.

### Induced motility experiments

Motility phenotype induction was assayed using soft-agar (0.25 %) plates with varying media compositions and supplements. Soft-agar plates were set up as described previously [38]. For induction of the *rhaSR*-P*rhaBAD* RpoN-EBP expression constructs, l-rhamnose was added to the motility agar with a final concentration of 0.15 % w/v. Activating signals R-hydroxybutyrate (R-HB – for *hbcR*), or Glycine (Gly – for *gcsR*) were added to media at concentrations of 30 mM and 10 mM respectively. Six biological replicates of each strain of interest were inoculated into the motility plates by picking single colonies with a sterile toothpick and stabbing into the soft agar. These replicates allow us to discern whether any motility is adaptive, as if all six replicates display the same phenotype it is unlikely that any mutations have occurred.

## Statistical analysis

All statistical analysis and data handling was performed using R core statistical packages. Differences between the rhamnose absent and present conditions were tested via two-way ANOVA, and differences between the two conditions for individual RpoN-EBPs tested using Wilcoxon tests. For differences in rewiring pathway frequency between rhamnose absent and present conditions, a test was used.

## Results

### Overexpression of RpoN-EBPs does not result in immediate rescue of motility in nearly all cases

We constructed rhamnose inducible expression systems for 11 *P *. *fluorescens* SBW25 RpoN-EBPs genes selected based on structural similarity to the FleQ protein, along with including representatives of orphan regulators (those lacking a cognate kinase) and members of two-component systems [33] – *aauR* (PFLU1134), *algB* (PFLU0088)*, fleR* (PFLU4441)*,* PFLU2055, *PFLU1132, dctD* (*PFLU0286*), *prpR* (*PFLU2386*), *hbcR* (*PFLU2630*)*, gcsR* (*PFLU4895*), *mifR* (*PFLU4954*), *PFLU2695* and *PFLU2209*. These expression constructs were introduced into the *P. fluorescens* SBW25 Δ*fleQ*Δ*ntrBC* genetic background. This background lacks both the FleQ flagellar regulator, as well as the highly evolvable NtrBC system studied previously [16, 34, 35] – preventing the presence of this dominant rewiring pathway from masking the effect of RpoN-EBP expression on evolutionary rescue of motility. Introduction of expression constructs was performed as single chromosomal insertions on a miniTn7 transposon. The native gene copies of the RpoN-EBPs were not deleted from the chromosome so this insertion results in effective duplication of the RpoN-EBP gene in question. This allows conservation of the native regulatory connections of each RpoN-EBP – the native gene copy maintains its promoter, terminator, operon structure and upstream cis-regulatory binding sites – with the expression construct copy acting to increase the concentration of the RpoN-EBP available within a cell without significantly impacting these GRN connections. Overexpression of each RpoN-EBP may however alter the abundance of GRN components via increased expression of downstream genes regulated by the relevant transcription factor. The fact that our constructs constitute duplications of the RpoN-EBP genes may impact the likelihood of their mutation. Providing a redundant copy may release pressure to maintain active function of the RpoN-EBP and can also restrict mutational availability through the suppression of recessive-effect mutations [47].

We began by testing whether increasing the expression of each RpoN-EBP resulted in immediate restoration of motility. Constructs were incubated in M9 motility agar supplemented with 0.15 % w/v l-rhamnose to induce expression. In almost all cases this did not result in immediate rescue of motility within 24 h. The exception was overexpression of *fleR* – an existing part of the flagellar regulatory cascade – which resulted in immediate flagellar motility (Fig. S3A, available in the online version of this article). This was curious, as there is no known mechanism for FleR to regulate the entire flagellar cascade by itself without the action of FleQ [48–50], which may suggest a previously undiscovered regulatory connection (Fig. S3B).

### Overexpression significantly increases the evolutionary rate of motility rescue for 5/11 RpoN-EBPs

**Fig. 1.**
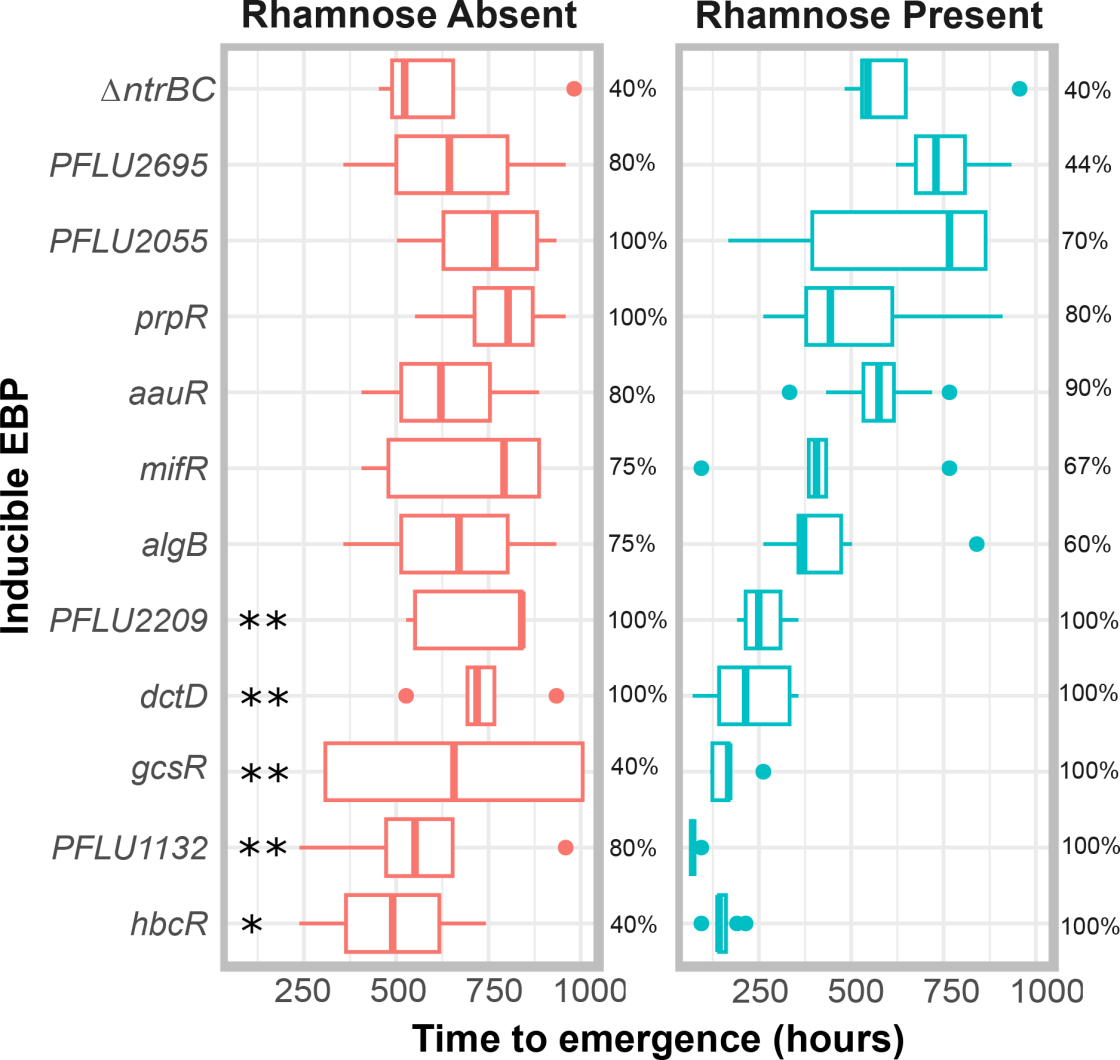
Time to emergence (TTE, hours) of flagellar motility in soft agar plates for each inducible RpoN-EBP system and the Δ*ntrBC* genetic background in the absence and presence of l-rhamnose (0.15 % w/v). Boxplots display median, and upper and lower quartiles in standard format. Statistically significant differences in time to emergence are indicated by *’s with * = 0.005 < *P* < 0.05; ** = *P* < 0.005 (Wilcox test). To the right of each boxplot, a percentage is given indicating the proportion of independent replicate plates that evolved motility within 6 weeks. Ten independent replicate evolution experiments were set up for each RpoN-EBP, with and without l-rhamnose.

To test if overexpression of each RpoN-EBP altered the mutational pathway for evolutionary rescue of motility, we incubated each expression construct in M9 motility agar with or without 0.15 % l-rhamnose supplement. We incubated plates for up to 6 weeks to allow motility mutants to evolve. In the Δ*fleQ*Δ*ntrBC* genetic background, the expected pathway of motility rescue is through the previously studied PFLU1131/2 two-component system [2]. We measured the time taken for the evolution of a motile phenotype (termed ‘time to emergence’) in this background, as well as for each RpoN-EBP expression construct in the presence and absence of l-rhamnose. The Δ*fleQ*Δ*ntrBC* control background evolved with an average time to emergence that was not significantly different with or without l-rhamnose supplement (632 and 620 h respectively, *P*=0.8824 Wilcox test). Broken down by each RpoN-EBP expression system, pairwise comparisons (Wilcox test) indicate no significant difference between presence and absence of l-rhamnose on time to emergence for *aauR, algB*, *PFLU2055*, PFLU2695, *mifR*, and *prpR*, but significant differences for *dctD*, *hbcR*, PFLU1132, PFLU2209, and *gcsR* (Fig. 1) . However, the addition of l-rhamnose significantly reduced the time to emergence of motility when all RpoN-EBPs are grouped, with mean time to emergence being 356 and 674 h respectively (Two-way ANOVA: F=2.07e-12; *P*<0.001), this indicates a strong effect of a negative correlation between TF expression and time for motility mutants to emerge despite gene specific responses to rhamnose.

### Overexpression of RpoN-EBPs results in switch of primary mutational targets, suggesting use of alternative evolutionary rewiring pathways

**Fig. 2.**
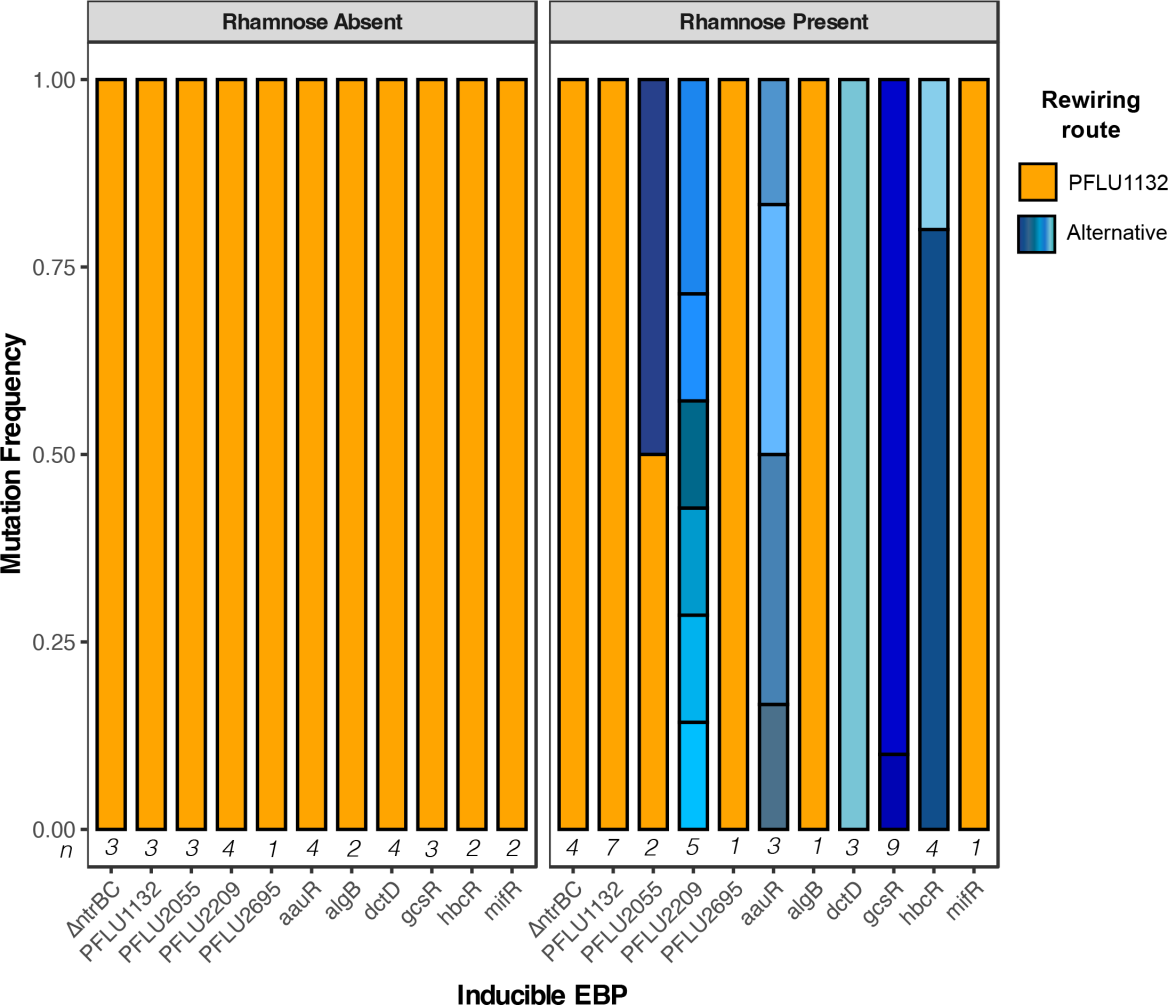
Proportion of observed mutations in evolved motile isolates. If a mutation was present in the PFLU1132 operon rewiring was assumed via this route, otherwise it is listed as an ‘alternative’ rewiring route. Mutations in the absence (left) and presence (right) of l-rhamnose are shown in panels. The number of each independent RpoN-EBP expression lines that evolved motility and were subsequently sequenced in each experiment are given below each bar (**n**).

To identify whether increased RpoN-EBP expression had an effect on mutational targets utilised for evolutionary rescue of flagellar motility, we performed whole genome resequencing on isolates that evolved motility within the 6 week time frame for each evolution experiment. In the no rhamnose condition, the primary mutational target was the PFLU1130/1/2 operon, with mutations in the PFLU1130/1/2 locus being present in 100 % of motile isolates for all RpoN-EBP expression strains tested in this media condition, as well as the *ΔfleQΔntrBC* control line (Fig. 2, Table S1). This was expected, as previous work identified this route as the primary pathway for evolutionary rescue of motility in the absence of *ntrBC,* where PFLU1131 mutation results in increased promiscuous regulatory activity in PFLU1132 and rescued flagellar gene expression [2]. Many isolates had additional mutations alongside those in the PFLU1132 pathway, which are detailed in full in Table S1. Two replicates in the no rhamnose control lines, one in an inducible *dctD* construct and one in an inducible *PFLU2209* construct, had mutations to *algB* alongside mutations in the PFLU1132 pathway – AlgB is an RpoN-EBP and FleQ homolog, so this may indicate involvement of this protein in rescuing motility in these isolates.

With addition of 0.15 % w/v l-rhamnose to the soft agar, mutations conferring motility within the PFLU1132 pathway became far less frequent, with the total number of isolates with PFLU1132 mutations across all test RpoN-EBP expression conditions (excluding *ΔfleQΔntrB* background and an RpoN-EBP expression strain for PFLU1132 itself – these control conditions are expected to gain mutations in PFLU1132 pathway) significantly dropping from 26/26 in the no-rhamnose control to 4/36 in the rhamnose condition ( $\chi^{2}$ : *P*=2.861e-11), with isolates from the *hbcR, aauR, gcsR, dctD* and *PFLU2209* overexpression conditions lacking any mutations in the PFLU1132 pathway. For the *ΔfleQΔntrBC* genetic background and PFLU1132 expression control lines, all motility rescue mutations in the presence and absence of l-rhamnose occurred in the PFLU1132 pathway (Fig. 2). When mutations occurred in the PFLU1132 gene itself, these occurred in the gene copy on the rhamnose inducible expression system, not the native chromosomal copy (Table S1).

**Fig. 3.**
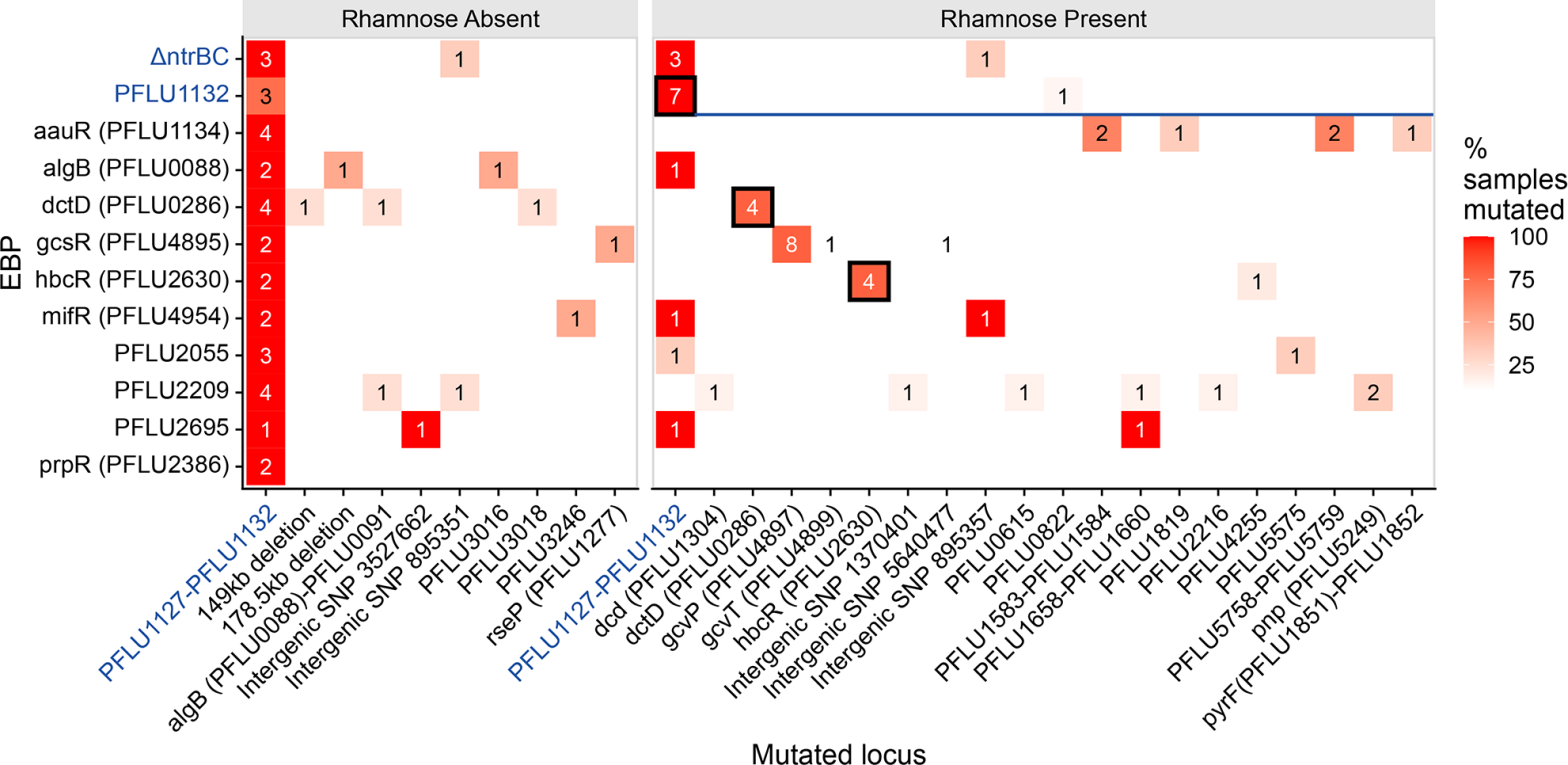
Counts of number of lines in which a genetic locus is mutated during motility rescue experiment for each RpoN-EBP expression line. Each panel is shaded by what percentage of the isolates in each condition have a mutation in that locus, e.g. where 100 % of lines contain the same mutation, the box is coloured bright red. Note, some isolates have more than one mutation. Where the mutation is within a copy of overexpressed RpoN-EBP, the box has a black border. The primary ‘expected’ PFLU1132 pathway is indicated by a blue label on the x axis. The left panel shows the locus that was mutated in the motile isolates evolved in the absence of l-rhamnose, the right panel shows mutated loci in the motile isolates evolved in the presence of l-rhamnose. Δ*ntrBC* and PFLU1132 are indicated by a blue label on the y axis and separator in the right panel, as they are expected to use the PFLU1132 pathway in the presence of l-rhamnose.

In some cases, motile isolates evolved in the presence of l-rhamnose instead gained mutations associated with the relevant overexpressed RpoN-EBP. These could be grouped in to two broad categories. The first are those RpoN-EBP expression strains for which the primary mutation target switched to being one of the two copies of the RpoN-EBP being overexpressed. Overexpression of *hbcR*, *dctD* both resulted in rescue of motility predominantly with mutations to these transcription factor genes (Fig. 3). Mutations in *hbcR* and *dctD* occurred in both chromosomal and inducible expression system copies of these genes at equal frequencies (Table S1). These mutations occurred in inter-domain regions of the RpoN-EBP proteins for most cases, aside from four which all occurred within the response receiver domain (Table S3). The second group do not gain mutations directly in the rhamnose-induced RpoN-EBP. These isolates instead gain mutations in a set of metabolic genes, which likely involve indirect modulation of RpoN-EBP activity through feedback.

The clearest example is that of *gcsR* overexpression, for which 8/10 motile isolates had gained a mutation in *gcvP*, one in *gcvT* and one in the intergenic region between PFLU5143 and PFLU5144. Of the eight *gcvP* mutations, four result in frame shifts, suggesting loss of function of the protein product. These genes encode the GcvP glycine dehydrogenase and the GcvT aminomethyltransferase, both components of the glycine cleavage system catabolic cycle [51, 52]. Loss of these catabolic enzymes will result in an accumulation of intracellular glycine, as seen previously for *gcvP* mutants in other organisms [53]. As GscR is a glycine responsive transcription factor [52], these mutations likely act to generate activating conditions for GcsR within the cytosol. Similarly, overexpression of *aauR* shifted the mutational spectrum to include genes involved in acidic amino acid metabolism. Across three motile isolates from the *aauR* condition there were individual cases of mutations in the genes *pyrB* (PFLU5758), *pyrC* (PFLU5759) and *pyrF* (PFLU1851). These genes encode enzymes aspartate carbamoyltransferase, dihydroorotase, and orotidine 5'-phosphate decarboxylase respectively, which are involved in the biosynthesis of pyrimidine nucleotides by converting aspartate to uridine-monophosphate [54, 55]. Motile isolates from the *aauR* overexpression condition gained INDELs in these genes, which likely inactivated these enzymes (in the case of *pyrF*, a large portion of the open reading frame was deleted) – again this likely causes an increase of intracellular aspartate, by which AauR activity is determined through its sensor-kinase AauS [56]. This would then generate conditions that activate the RpoN-EBP AauR in the cytosol. Interestingly, alongside mutations affecting aspartate catabolism, two motile *aauR* overexpression isolates had mutations in PFLU1583 and PFLU1584. These genes encode a putative anti-sigma factor system, which we have previously shown to facilitate RpoN-EBP promiscuity of PFLU1132 [2]. This suggests that mutations to PFLU1583/4 may constitute a general mechanism of enhancing promiscuity in RpoN-EBPs, as here mutations to this system are present in the absence of any PFLU1131/2 mutation. Finally, overexpression of *PFLU2055* and *PFLU2209* also resulted in motile isolates with putative feedback mutations. One *PFLU2055* motile isolate had a mutation to *lptD* (an LPS-assembly enzyme) – which may impact cell membrane integrity, which PFLU2055 homolog, PspF, has been shown to respond to [57]. For PFLU2209, whilst its native function is unknown, mutations in motile isolates included genes involved in nucleotide metabolism (*pnp*, *dcd*).

Overexpression of these RpoN-EBPs resulted in significant shifts in mutational targets associated with rescue of motility, away from the primary PFLU1132 pathway utilised in these conditions. Rescue of motility primarily preceded through mutations acting on the overexpressed RpoN-EBP in question – either with mutation directly to the RpoN-EBP gene (as seen in *hbcR* and *dctD*), or the genes associated with its regulatory function, as was the case for *gcsR* and *aauR*. These results indicate that many members of the RpoN-EBP family of transcription factors are capable of rewiring to rescue motility in our assay, and that the expression of the RpoN-EBP is a significant factor constraining their potential to evolve novel interactions (Fig. 3).

### Flagellar motility can be immediately rescued without mutation if an RpoN-EBP is overexpressed and activating environmental signals are present

We previously discussed the possibility that environmental conditions may ‘prime’ transcription factors for rewiring by providing conditions of high activation [3].

Our experiments demonstrated that for some cases, an RpoN-EBP that was overexpressed could rescue motility through mutations that likely yielded increased cytosolic concentrations of the signal to which the RpoN-EBP in question responded (e.g. overexpression of glycine-responsive regulator *gcsR* resulted in mutations in the glycine cleavage pathway). Such activating signals could also feasibly be provided by the external environment rather than mutation to internal metabolism. To test this, we selected two RpoN-EBPs for which an activating signal could be provided externally – *gcsR*, and *hbcR*. This pair were chosen as contrasting examples – *gcsR* rescued motility in our experiment through mutations to internal glycine catabolism, whereas *hbcR* evolved through mutation to one of the two copies of the RpoN-EBP gene itself. However, the HbcR transcription factor can be activated by the presence of (R)−3-hydroxybutyrate (R-HB), a metabolite which cannot be produced by internal metabolism [58]. This may explain why in our assay, mutation to *hbcR* itself are the primary mechanism of rescuing motility for this RpoN-EBP, as higher levels of R-HB cannot be produced internally.

**Fig. 4.**
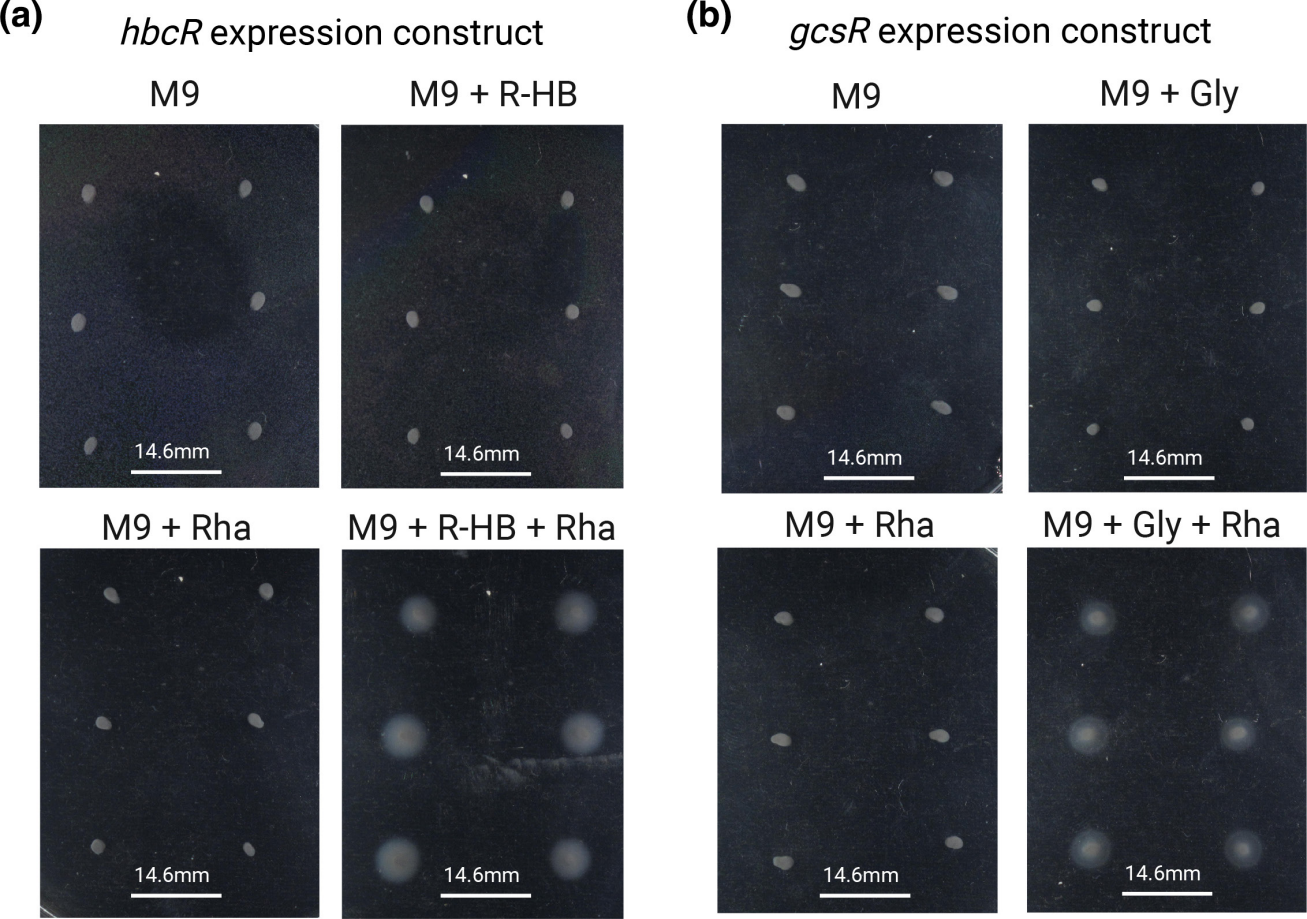
Flagellar motility can be rescued by alternative RpoN-EBPs if overexpressed and in the presence of an activating signal. **a**) *hbcR* and b) *gcsR* after 24 h of incubation in 0.25 % agar motility plates. Plates consisted of M9 media, with either no supplement, 0.15 % l-rhamnose (+Rha), an environmental signal for each RpoN-EBP (30 mM R-hydroxybutyrate (+R-HB) for *hbcR*, or 10 mM Glycine (+Gly) for *gcsR*), or both signal and l-rhamnose.

To test if flagellar motility could be rescued by environmentally providing activating signals for these two transcription factors, we set up M9 soft agar plates supplemented with l-glycine for *gcsR* or R-HB for *hbcR*. In both cases, incubation of these strains in the presence of both l-rhamnose (to ensure RpoN-EBP overexpression), and the relevant activating signal resulted in immediate flagellar motility (Fig. 4). We additionally demonstrated that activating signals that facilitate rewiring can be provided environmentally by overexpressing the *ntrBC* two component system in the presence of glutamate, which resulted in immediate motility – curiously this did not result in motility when only *ntrC* was overexpressed – which likely indicates the importance of TCS stoichiometry (Fig. S4).

To confirm that motility depends on overexpression of these RpoN-EBPs and their interaction with their respective signals, control plates of the rhamnose-inducible expression system lacking any RpoN-EBP, and of the *mifR* overexpression strain (RpoN-EBP overexpressed should not respond to either signal) were run (Fig. S5) and were found to remain immotile upon addition of l-rhamnose, R-HB and l-glycine in the relevant combinations. This confirms that rescue of flagellar motility via rewiring of RpoN-EBP transcription factors can also be facilitated by presence of signals that activate the relevant transcription factor in the environment.

## Discussion

Through manipulation of transcription factor expression, we have identified several novel pathways for evolutionary rescue flagellar mediated motility in *P. fluorescens *, in addition to those that have been previously capable of innovation to rescue motility without modified expression (NtrC and PFLU1132) [2, 16]. Regulators HbcR, AauR, GcsR, DctD, and PFLU2209 all became viable evolutionary routes upon induced high expression, either via mutation to the transcription factor itself (HbcR, DctD), or via physiological feedback (HbcR, GcsR, PFLU2209). These results lend significant strength to our prior hypothesis that the expression of a transcription factor (and by extension its connectivity within the gene regulatory network) will constrain its ability to gain promiscuous activity and innovate novel regulatory functions [2]. Additionally, we have previously suggested that hyperactivation of a transcription factor is important for innovation [2], and again our results support this – HbcR, GcsR and AauR have all been shown to be capable of rescuing motility through production (or external supply) of an activating metabolic signal. That transcription factor expression can have such an impact on the availability of evolutionary trajectories has significant implications for regulatory evolution and innovation – suggesting that transcription factor expression and activity (which are themselves determined by GRN architecture and environmental signals) have the potential to determine the prevailing adaptive outcome.

This set of identified transcription factors capable of rescuing flagellar motility, together with NtrC and PFLU1132, constitute 58 % of the RpoN-EBP transcription factor family tested by our work, and 33 % of the total encoded by *Pseudomonas fluorescens* SBW25. For all these regulators, no canonical regulatory link to the flagellum is known, yet flagellar expression is rescued through a small number of mutational steps when these RpoN-EBPs are overexpressed. Why overexpression of the remaining regulators (*mifR*, *algB* and PFLU2695) do not alter the evolutionary pathway for rescue of flagella motility is unknown, however could be due to differences in the precise expression levels of these RpoN-EBPs, or local GRN connections suppressing activity or not providing a suitable regulatory link to the flagellar genes.

The fact that so many members of the RpoN-EBP transcription factor complement in *P. fluorescens* SBW25 can rescue flagellar motility suggests that this transcription factor family may be uniquely suited to regulatory innovation. Why this should be the case is unclear, however the answer may lie in the unique regulatory mechanism of the RpoN-EBP family. The sigma factor, RpoN, plays a pivotal role in localising the EBP so that it may provide ATPase activity to catalyse transcription initiation [59]. RpoN-EBPs are indeed a highly diverse and varied class of transcription factors [60], and numbers of RpoN-EBPs encoded can significantly vary between bacteria – *P. fluorescens* encodes ~20*, Escherichia coli* ~12, *Bacillus subtilis* ~5, *Chlamydia pneumoniae* and *Treponema pallidum* both encode one each, and *Mycoplasma genitalium* and *Rickettsia prowazekii* encode none [61]. Even within a single order or genus, the number can be highly variable, with a range of 1–35 RpoN-EBPs present in 57 *Clostridiales* species [62], and between 9–29 RpoN-EBPs within the *Pseudomonas* genus (see Methods, Table S4). It has been suggested previously that RpoN-EBPs provide a biological advantage in regulating genes compared to other regulators, potentially due to providing ‘leaky’ regulation [61]. This can be an advantage in changeable environments [63], situations also evidenced to drive regulatory innovation within GRNs [64]. Our results indicate that this family of regulators may be well suited toward regulatory innovation, a key factor in coping with changeable conditions. Additionally, our findings suggest that possessing large sets of transcription factors derived from the same protein family may increase the opportunity for promiscuity and innovation to occur via alteration to interconnectivity between similar regulatory proteins.

Alongside showcasing the ability of a set of RpoN-EBPs to rewire when overexpressed, we have also demonstrated that signals that activate transcription factors can also aid innovation. The nature of connections within regulatory networks will not only determine which transcription factors are highly expressed but also which are activated at any given moment via transduction of environmental signals [65, 66]. Both the pre-existing expression levels of a transcription factor, and the prevailing environmental conditions both therefore have the potential to influence which transcriptional regulators are evolvable. We have previously suggested that environmental conditions may ‘prime’ particular transcription factors for evolutionary innovation [3]. Our results in this work support this suggestion, as we demonstrate that activating signals can result in rescued flagellar motility when present in combination with increased transcription factor expression for regulators HbcR and GcsR.

Our data also raise the intriguing possibility that transcription factors that are responsive to signals which can be generated via mutation to internal cellular physiology, may more easily evolve novel phenotypes than those than respond to purely external signals. In the case of AauR and GcsR, mutations occurred in metabolic pathways that catabolise the chemical signals to which these regulators respond (Aspartate and Glycine), likely resulting in their activation. Similarly, in our previous studies of NtrBC rewiring, the *glnA* gene encoding glutamine synthetase was a possible mutational target [16, 34, 35]. Loss of function to GlnA generates a condition of low glutamine concentration within the cell, which triggers activation of NtrBC through GlnK [67]. This type of internal metabolic mutation that will yield a high concentration of an activating metabolite signal did not occur for HbcR – mutations primarily targeted the transcription factor itself – possibly because its activating signal, R-HB, cannot be generated by internal metabolism [58]. When R-HB was supplied externally alongside high HbcR expression, motility was restored. The ease by which such an activating signal is provided is therefore also important in shaping and constraining the ability of a transcription factor to evolve, alongside the expression level of that transcription factor. Regulators that respond to external physical conditions (e.g. light or temperature), or exogenous metabolites (e.g. R-HB) may therefore be less likely to engage in evolutionary innovation.

Our results indicate the key role that transcription factor expression can play in constraining avenues of evolutionary innovation and evolvability in their constituent transcription factors. We have demonstrated that by increasing the expression of a transcription factor – representing an altered GRN architecture – that transcription factor can become the preferred candidate for evolutionary rewiring in our model system. We have demonstrated propensity for rewiring in multiple RpoN-EBP family members and highlighted the interplay between GRN architecture and prevailing environmental conditions necessary to facilitate rewiring in certain transcription factors. Our findings help to build a systems-level understanding of how conditions and signalling within gene regulatory networks can influence the evolutionary trajectories of their constituent components and create or constrain opportunities for innovation of transcription factors.

## Supplementary Data

Supplementary material 1

Supplementary material 2

Supplementary material 3

Supplementary material 4
