# Supplementary material for: Transcription factor expression levels and environmental signals constrain transcription factor innovation
Source: Microbiology (Reading). 2023 Aug 16;169(8):001378. doi: 10.1099/mic.0.001378 (PMC10482368; doi:10.1099/mic.0.001378)
Supplement: Supplementary material 1 [file mic-169-1378-s001.pdf]

Supplementary Figure S1

A

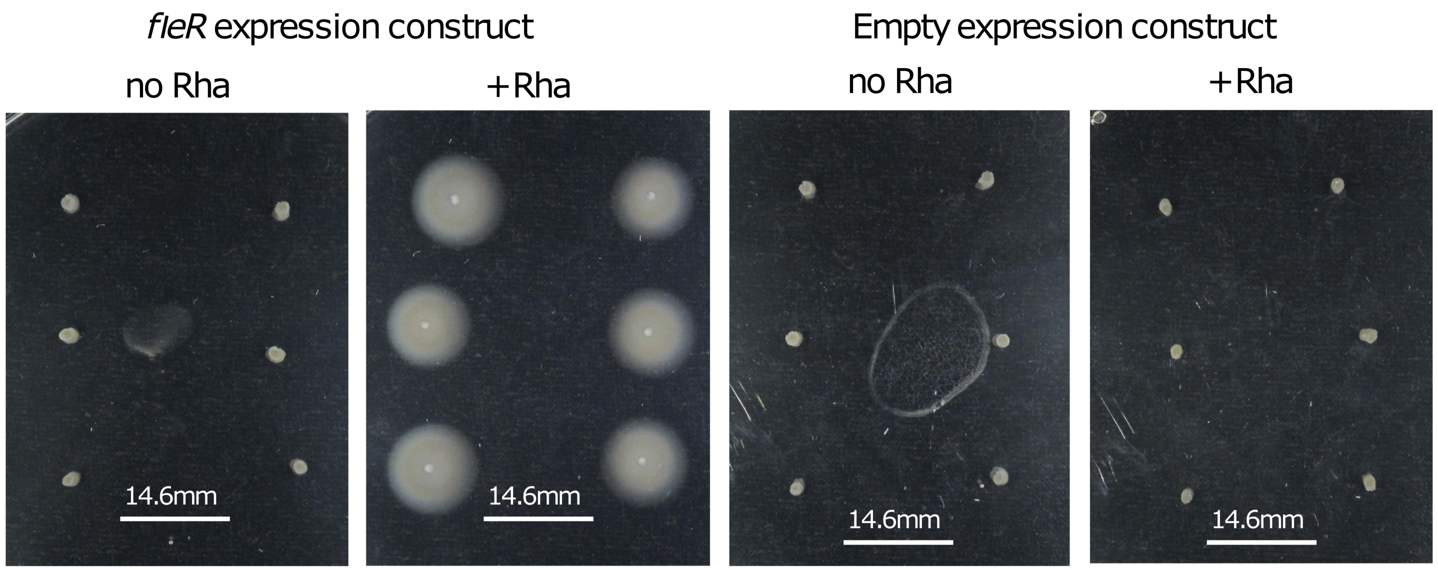

B

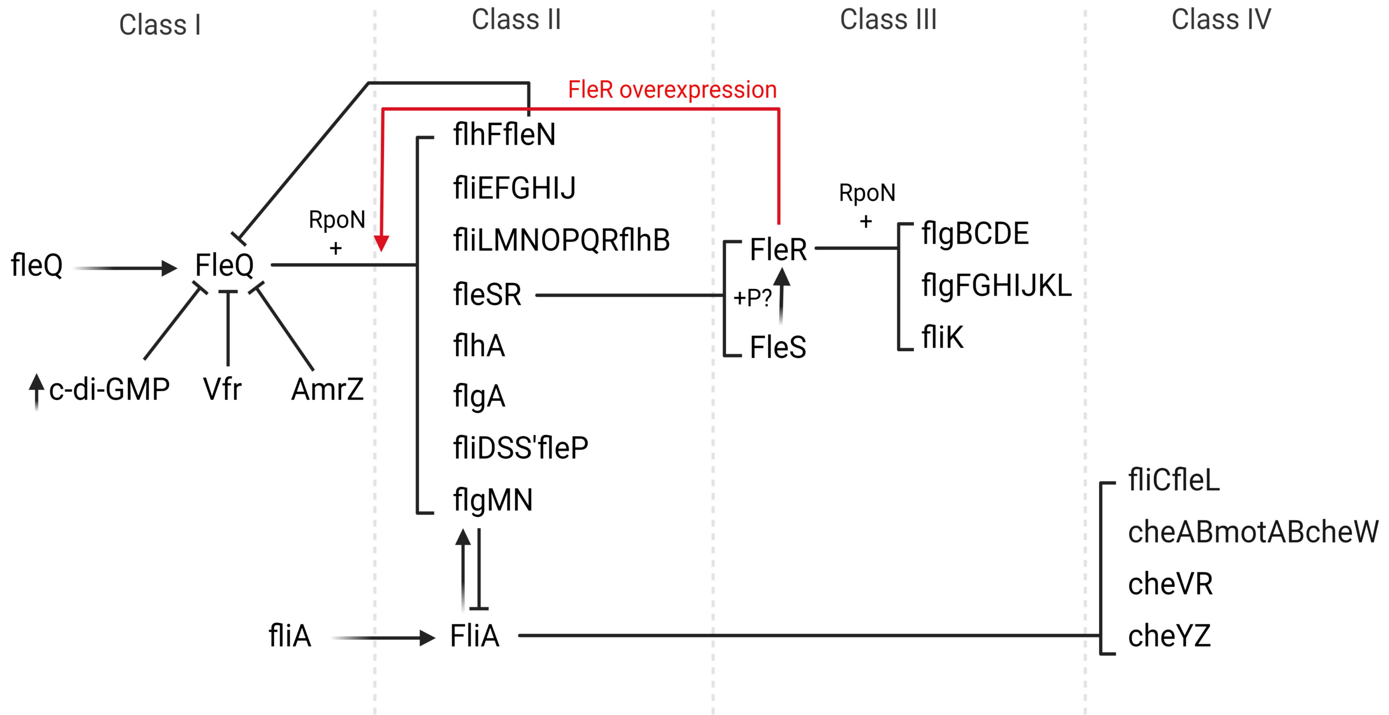

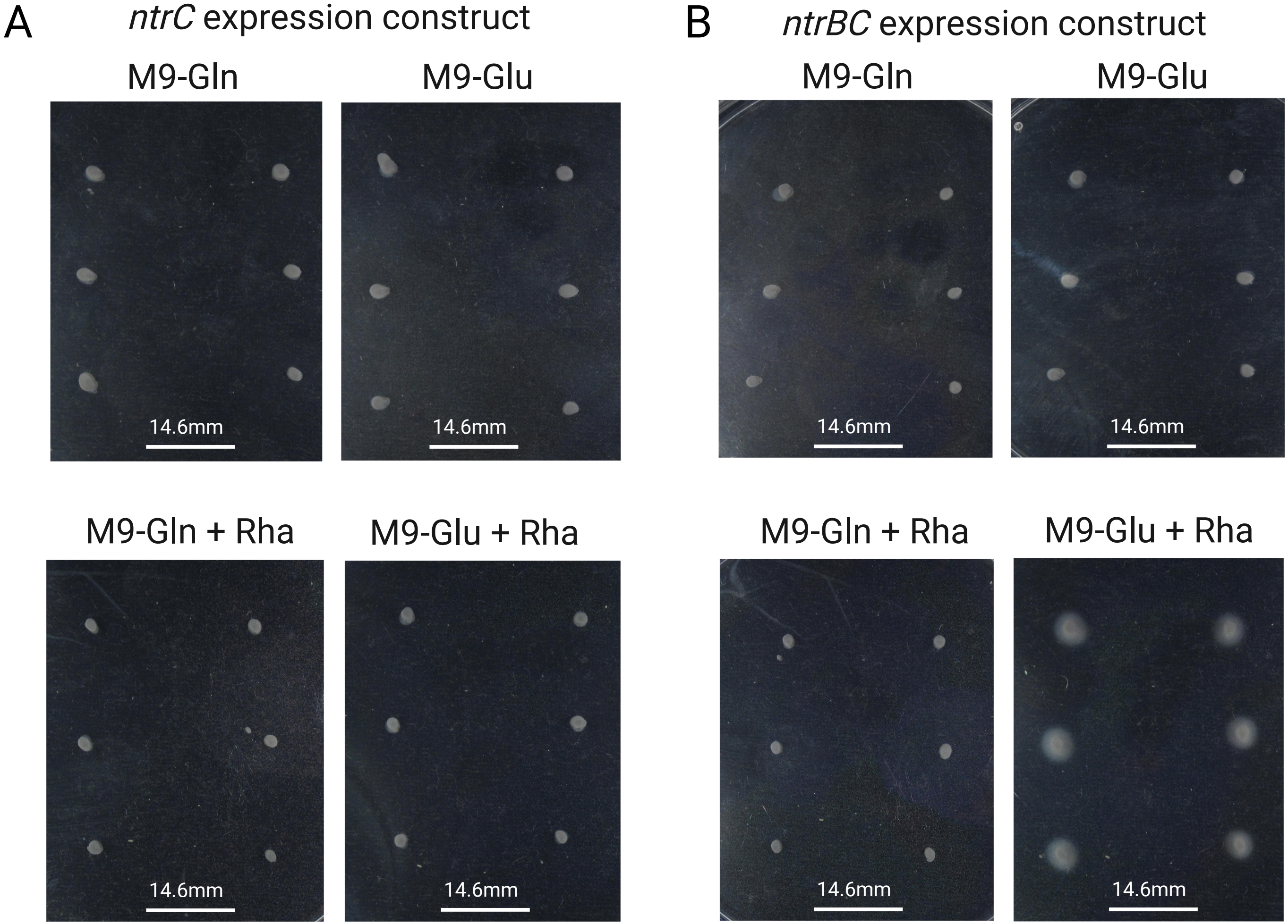

Supplementary Figure S2

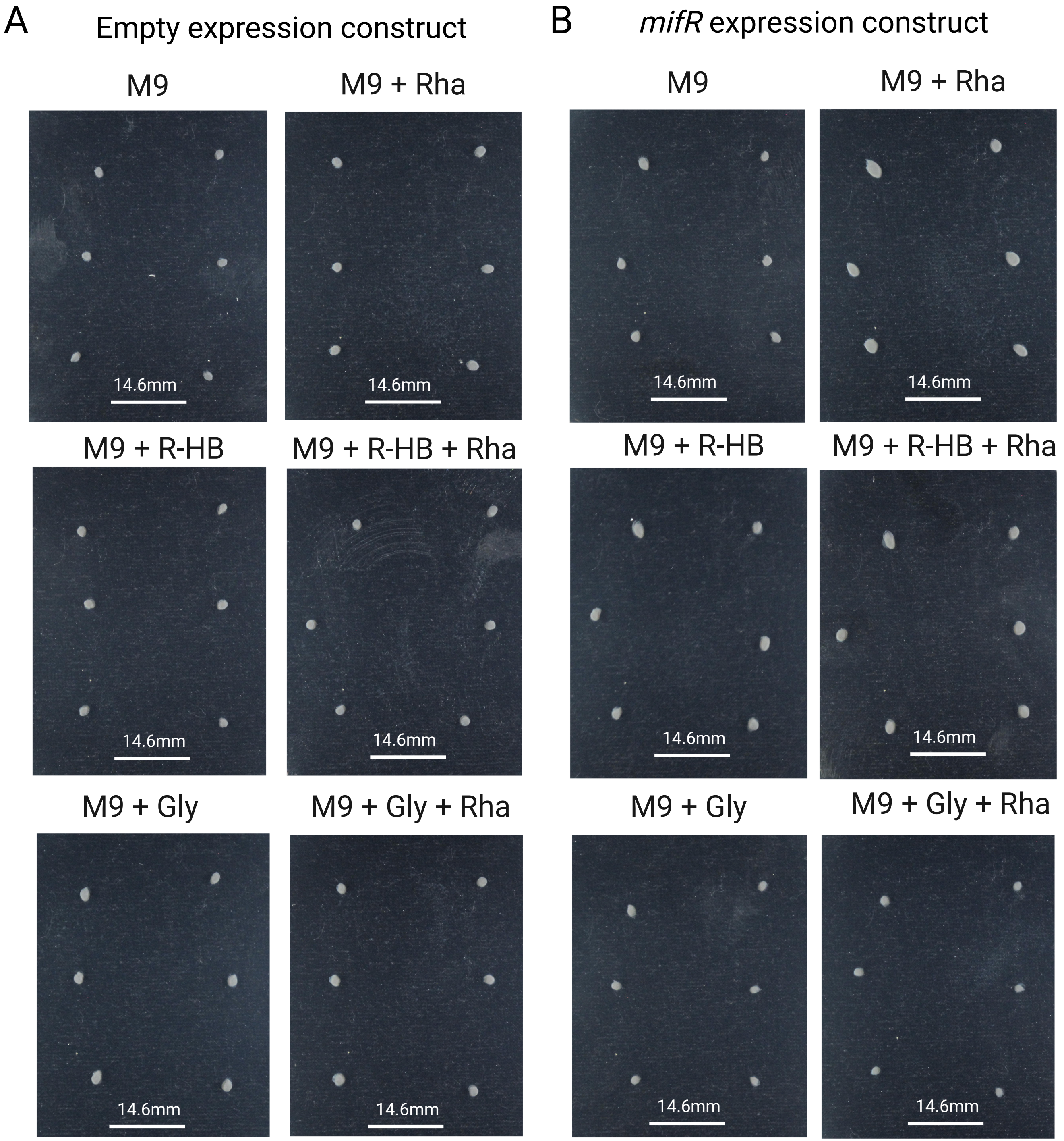

Supplementary Figure S3

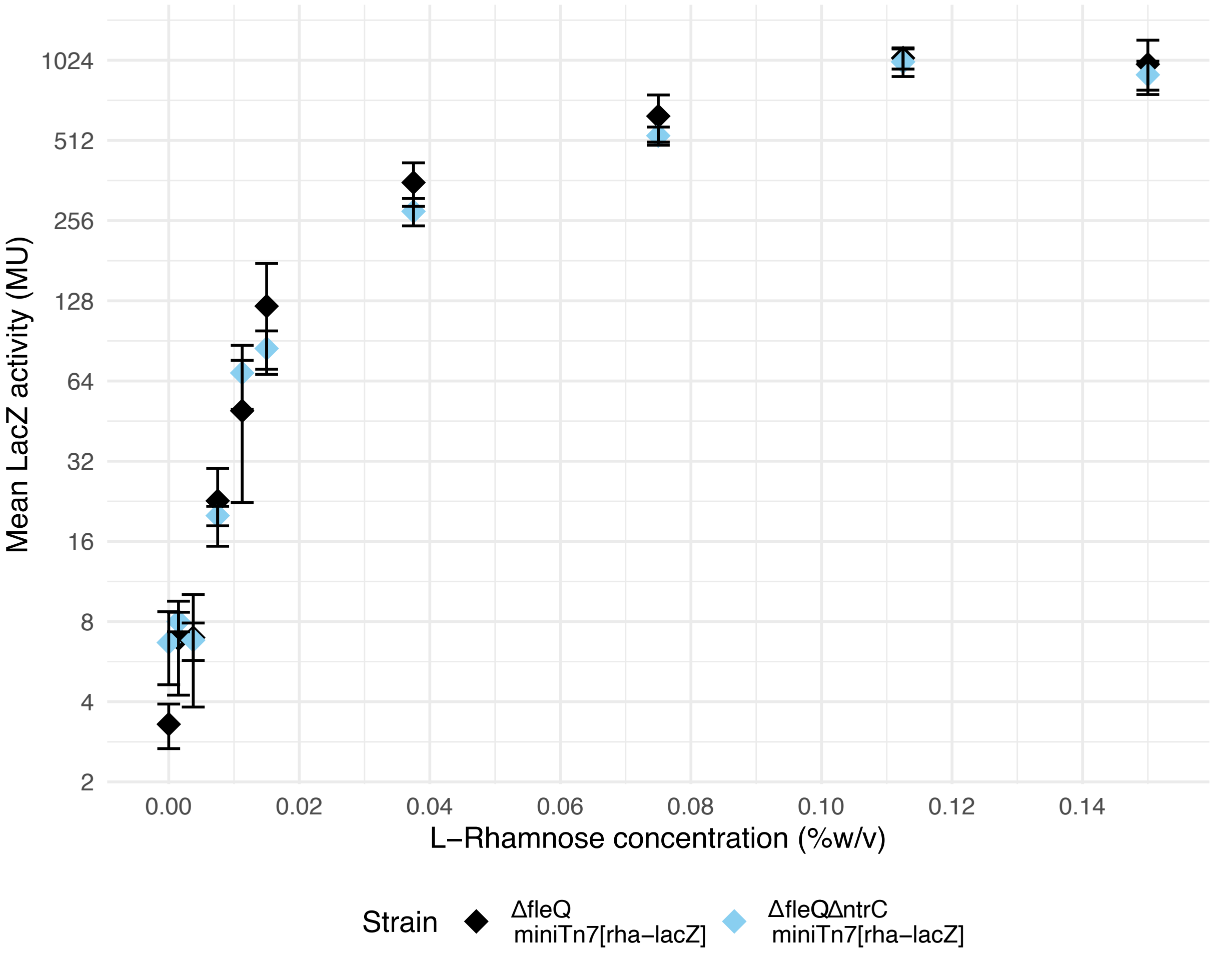

Supplementary Figure S4

Spearman's rho: ΔfleQ rha−lacZ = 0.981, ΔfleQΔntrC rha−lacZ = 0.955

A

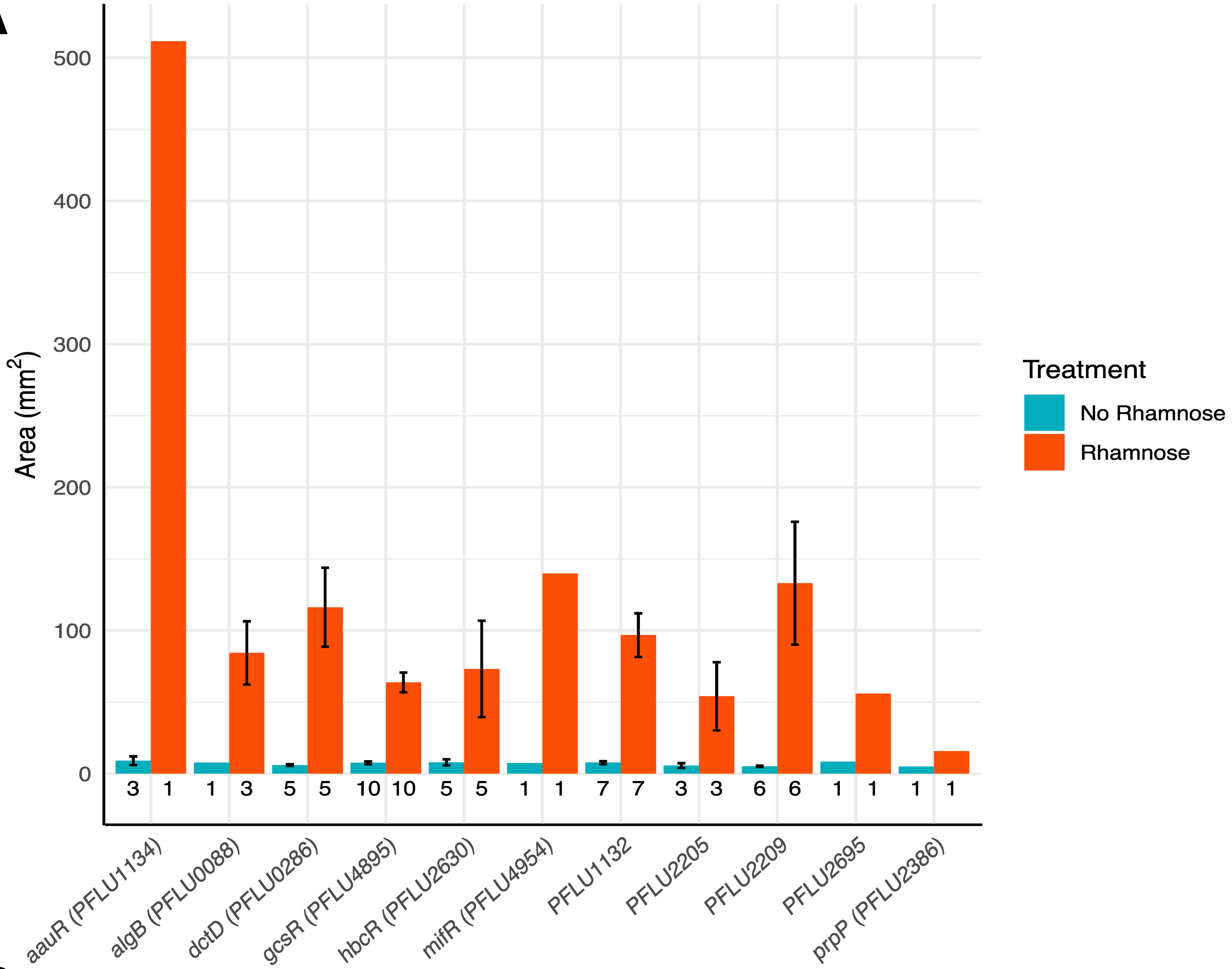

B

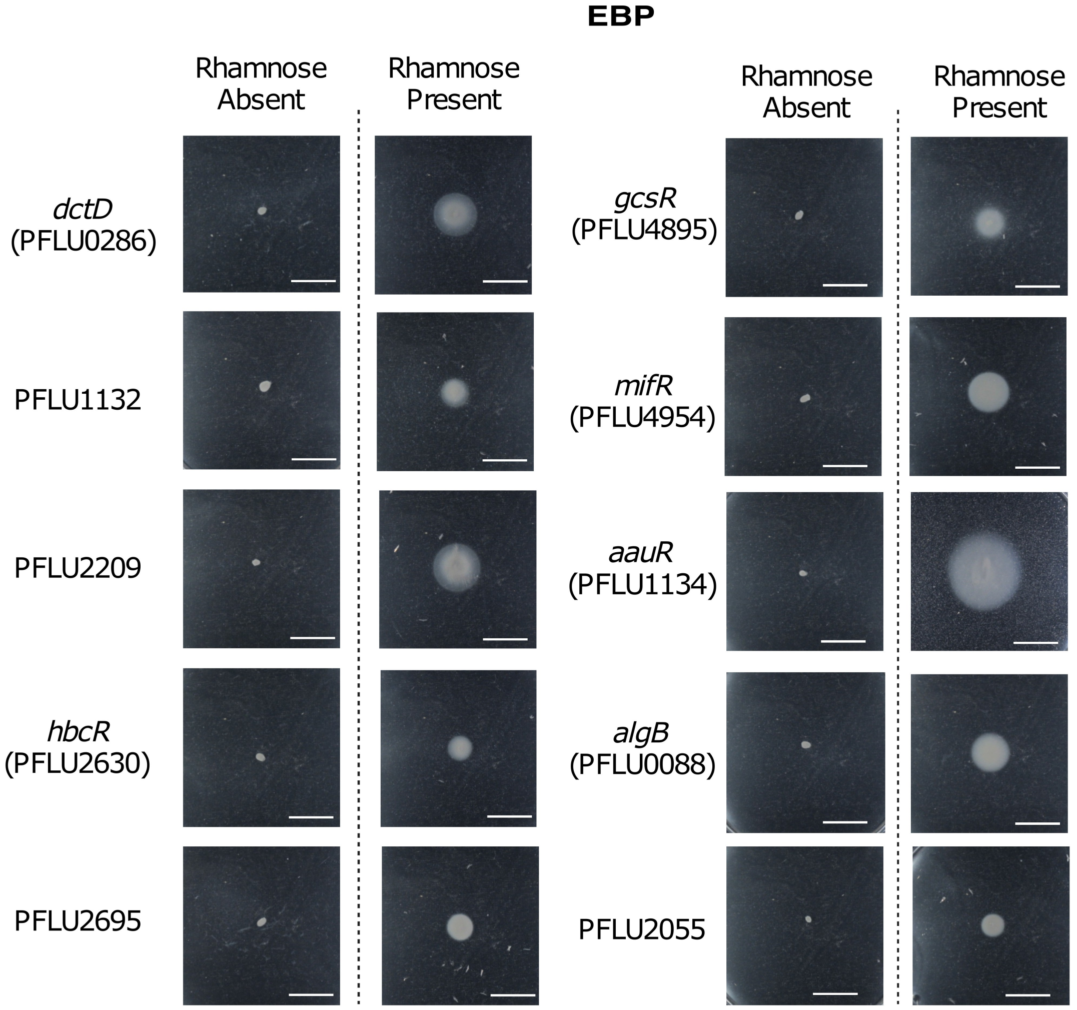

Supplementary Figure S5

Supplementary table S2:

Name	Sequence	Pair product size	Restriction site introduced
aauR-Forward	AATTTACTAGTTAAGGAGGAACAGCTATGAATACAGACGCTACAG	1387	SpeI
aauR-Reverse	AATTTAAGCTTTCAGTGGCTCAGTCCG		HindIII
algB-Forward	AATTTCTGCAGTAAGGAGGAACAGCTATGGAATCAGCCAAGGAAC	1384	PstI
algB-Reverse	AATTTAAGCTTTCACAGGTTGTACTGTTTGCG		HindIII
FleR-Forward	AATTTACTAGTTAAGGAGGAACAGCTATGGCTATCAAGGTTCTATTGG	1414	SpeI
FleR-Reverse	AATTTAAGCTTTTATGTGGCGAAAAGATACGC		HindIII
ntrC-Forward	AATTTCTGCAGTAAGGAGGAACAGCTATGAGCCGTAGTGAAACCG	1474	PstI
ntrC-Reverse	AATTTAAGCTTTCAGCCTTCATCGCCCTC		HindIII
PFLU0286-Forward	AATTTCTGCAGTAAGGAGGAACAGCTATGAGTATCGATAACCAGATTC	1414	PstI
PFLU0286-Reverse	AATTTAAGCTTTCATTCGTCGTCGCCAC		HindIII
PFLU2209-Forward	AATTTACTAGTTAAGGAGGAACAGCTTTGTCTATGAACATCCCGCG	1123	SpeI
PFLU2209-Reverse	AATTTAAGCTTTCAGGGTTTTGCTCGACC		HindIII
PFLU2386-Forward	AATTTCTGCAGTAAGGAGGAACAGCTAACATGAACATGGATACCG	2020	PstI
PFLU2386-Reverse	AATTTAAGCTTTAAATCTCAGTCGCCACG		HindIII
PFLU2630-Forward	AATTTCTGCAGTAAGGAGGAACAGCTATGAACATCACCGATAACCTC	1438	PstI
PFLU2630-Reverse	AATTTAAGCTTTCAGTCGATGCCCAGGG		HindIII
PFLU2695-Forward	AATTTACTAGTTAAGGAGGAACAGCTATGGGTGGACCCCCCGTTC	1363	SpeI
PFLU2695-Reverse	AATTTAAGCTTCTAGCGGATCTGGTGTTTG		HindIII
PFLU4895-Forward	AATTTACTAGTTAAGGAGGAACAGCTATGCGTATCCACGTCAG	1549	SpeI

PFLU4895-Reverse	AATTTAAGCTTCCAGTCAGGGCTTGTTGGAGGG		HindIII
PFLU4954-Forward	AATTTACTAGTTAAGGAGGAACAGCTATGCTGAACGCGGTGATTG	1363	SpeI
PFLU4954-Reverse	AATTTAAGCTTCTAGAGAAACATCTCCCGC		HindIII
PFLU2055-Forward	AATTTCTGCAGTAAGGAGGAACAGCTATGCAGCTTTTGACCCTACC	1141	PstI
PFLU2055-Reverse	AATTTAAGCTTCTAAATGGATAGATGCAACATG		HindIII
ntrBC-Forward	AATTTCTGCAGTAAGGAGGAACAGCTATGACCATCAGCGATGCACTGC	1437	PstI
ntrBC-Reverse	AATTTAAGCTTTCAGCCTTCATCGCCCTC		HindIII
pJM220 300bp Foward	GCGGTGAGCATCACATCACC	300bp if pJM220 MCS is empty	
pJM220 300bp Reverse	TAGATGGGAACTGGGTGTAGCG	300bp if pJM220 MCS is empty	
